# Alveolar macrophage - derived exosomes modulate severity and outcome of acute lung injury

**DOI:** 10.18632/aging.103010

**Published:** 2020-04-07

**Authors:** Cong Ye, Huiting Li, Minwei Bao, Ran Zhuo, Gening Jiang, Weixi Wang

**Affiliations:** 1 Department of Thoracic Surgery, Shanghai Pulmonary Hospital, Tongji University, Shanghai 200433, China; 2 Department of Respiratory Medicine, Shanghai Pulmonary Hospital, Tongji University, Shanghai 200433, China; 3 Department of Urology, Zhongshan Hospital, Fudan University, Shanghai 200032, China; 4 Department of Geriatrics, Zhongshan Hospital, Fudan University, Shanghai 200032, China

**Keywords:** acute lung injury (ALI), macrophages, neutrophils, exosomes, bronchoalveolar lavage fluid (BALF)

## Abstract

Severe acute lung injury (ALI) can cause death, and the survivals may develop acute respiratory distress syndrome (ARDS) due to fibrotic repair of the lung. Alveolar macrophages play a demonstrative role during the pathogenesis of ALI, and the timing and degree of differentially polarization of macrophages determine the severity of disease and outcome. Exosomes are important mediators of cellular communication and play critical roles during macrophage differentiation, proliferation and function. Nevertheless, the exact effects of alveolar macrophage - derived exosomes on ALI remain unknow. Here, we used lipopolysaccharide (LPS) to induce ALI in mice and analyzed the exosome population in bronchoalveolar lavage fluid (BALF) from macrophages, neutrophils and epithelial cells at different time points after treatment. Our data showed that macrophages were the major secretors for early secreted pro-inflammatory cytokines in the BALF-exosomes, which likely activated neutrophils to produce a variety of pro-inflammatory cytokines and IL-10. IL-10 by neutrophils in BALF-exosomes likely in turn polarized macrophages to M2c, which may be responsible for post-ALI fibrosis. Our study thus reveals a previous non-acknowledged role of BALF-exosomes as a mediator of inflammatory response and cell crosstalk during ALI.

## INTRODUCTION

Acute lung injury (ALI) is a common clinical lung disease, and can cause death, and the survivals may develop acute respiratory distress syndrome (ARDS) due to fibrotic repair of the lung. ARDS is a devastating clinical syndrome characterized by non-cardiogenic pulmonary edema, respiratory distress and hypoxemia [1]. Although the ARDS-associated mortality has decreased in the last decade, still about half of the patients die, while the survivors suffer from significant physical and psychological impairments [2–4].

Alveolar macrophages play a demonstrative role during the pathogenesis of ALI, and the timing and degree of differentially polarization of macrophages determine the severity of disease and outcome [5]. Exosomes are cell-secreted nanosized bi-lipid membrane in the secretome. Bidirectional communication occurs in microenvironment via exosomes and microvesicles (MVs) [6]. Exosomes and MVs carry nucleic acids, proteins, and lipids between different cells of the tumor microenvironment, which influence a multitude of pathways biologically. Exosomes are 30–100 nm in diameter and are generated within larger intracellular multivesicular bodies [6]. Exosomes and microvesicles (MVs) are released into the extracellular environment on fusion with the plasma membrane [6]. MVs generally range from 100 to 1000 nm and are formed when cell components travel to the plasma membrane to be released by membrane budding [6]. Actually, the previous studies did not strictly distinguish exosomes from MVs, from definition to isolation protocols. Exosomes, the validation of which includes expression of CD9, CD63, CD81, ALIX and TSG101, are important mediators for cellular communication and play critical roles during macrophage differentiation, proliferation and function [7]. Nevertheless, the exact effects of alveolar macrophage - derived exosomes on ALI remain unknow.

In this study, we used lipopolysaccharide (LPS) to induce ALI in mice and analyzed the exosome population in bronchoalveolar lavage fluid (BALF) from macrophages, neutrophils and epithelial cells at different time points after treatment. Our data showed that macrophages were the major secretors for early secreted pro-inflammatory cytokines in the BALF-exosomes, which likely activated neutrophils to produce a variety of pro-inflammatory cytokines and IL-10. IL-10 by neutrophils in BALF-exosomes likely in turn polarized macrophages to M2c, which may be responsible for post-ALI fibrosis.

## RESULTS

### Purification of BALF-derived exosomes from ALI-mice

**Figure 1:**
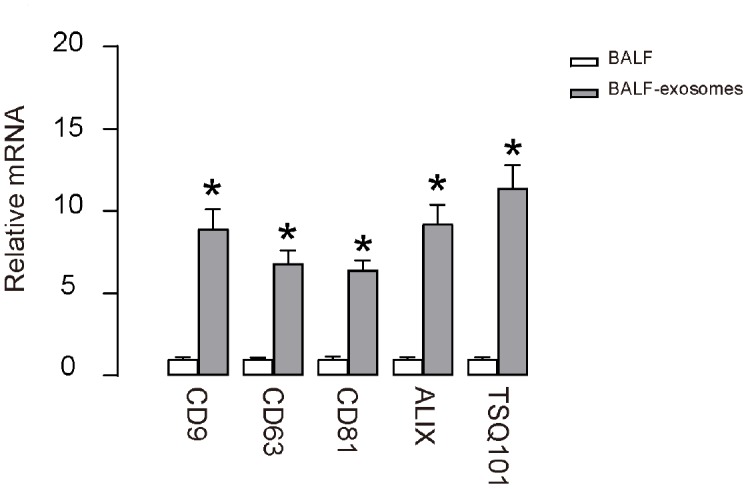
**Purification of BALF-derived exosomes from ALI-mice.** RT-qPCR for CD9, CD63, CD81, ALIX and TSQ101 in purified exosomes from BALF after ALI. *p<0.05. N=5.

ALI models were applied to mice and BALF were isolated at different time points. Exosomes were then purified from the prepared BALF, confirmed by enriched expression of CD9, CD63, CD81, ALIX and TSQ101 in exosomes, compared to the corresponding total BALF (Figure 1). Hence, exosomes were successfully isolated from ALI-BALF.

### Purification of macrophage-, epithelia- and neutrophil- derived exosomes from total BALF-exosomes from ALI-mice

**Figure 2:**
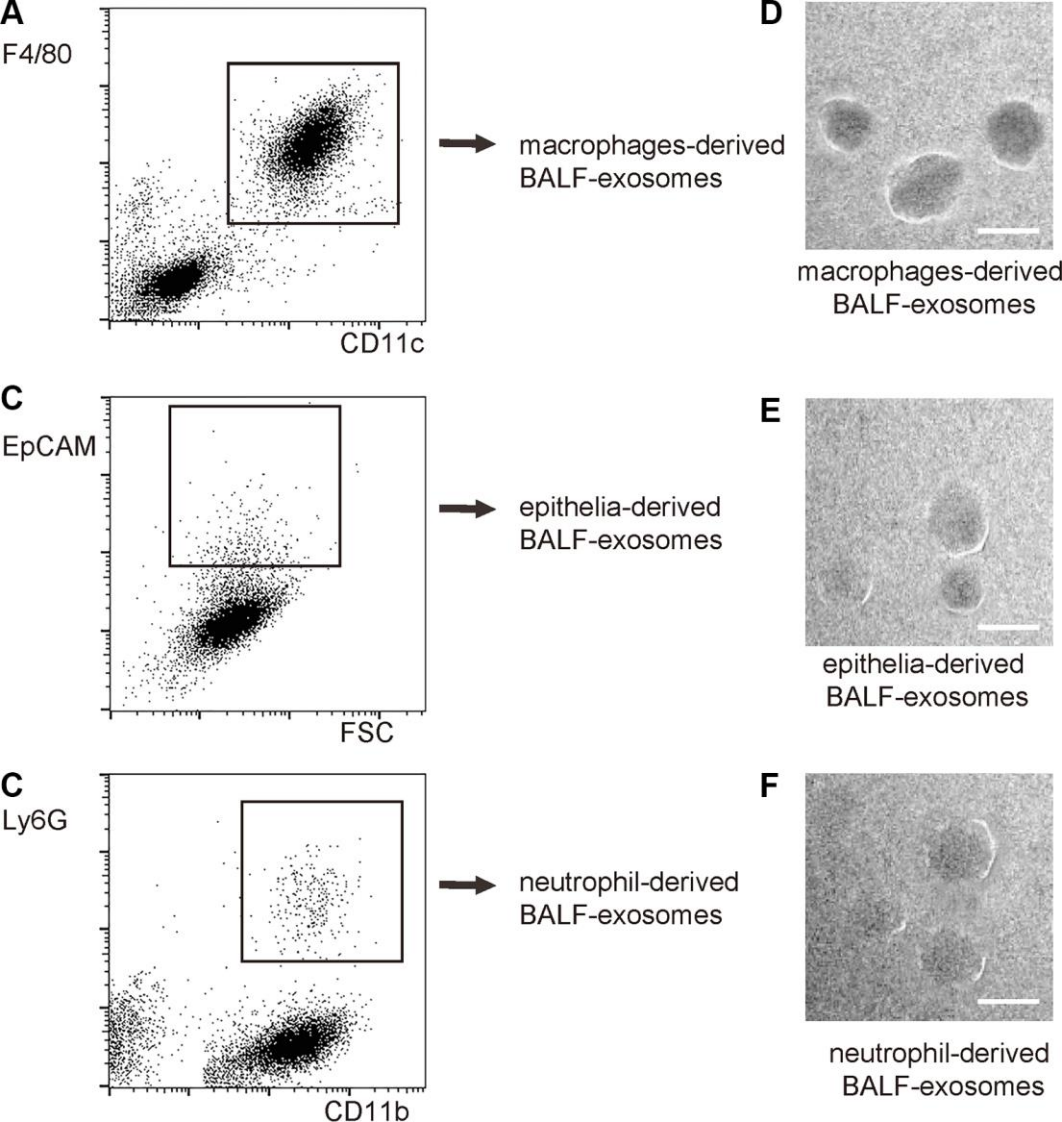
**Purification of macrophage-, epithelia- and neutrophil- derived exosomes from total BALF-exosomes from ALI-mice.** The isolated BALF-exosomes were further purified into macrophage-, epithelia- and neutrophil- derived exosomes, based on differential expression of precursor cell markers. The purification strategy included size less than 500nm and being positive for specific precursor cell markers. (**A**) Flow chart for macrophage-derived exosomes: positive for both CD11c and F4/80. (**B**) Flow chart for epithelia: positive for EpCAM. (**C**) Flow chart for neutrophils: positive for both CD11b and Ly6G. (**D**–**F**) EM visualization of exosomes purified from isolated exosomes. Scale bars are 50nm.

Next, we used precursor cell markers to purify exosomes derived from macrophages, epithelia and neutrophils, since these three cell types are the major cell types that contribute to the cytokine production and secretion after ALI. The exosomes not from these three cell types could be derived from T-cells, endothelial cells and other cell types. The purification strategy included size less than 500nm and being positive for specific precursor cell markers. Macrophages: positive for both CD11c and F4/80 (Figure 2A). Epithelia: positive for EpCAM (Figure 2B). Neutrophils: positive for both CD11b and Ly6G (Figure 2C). Purified exosomes could be visualized under electron microscopy (Figure 2D–2F). Using this methodology, the dynamics of exosome release from macrophages, epithelial cells and neutrophils within the alveolar space were determined during the different phase (1 hour, 4 hours, 8 hours, 16 hours and 32 hours) of LPS-induced ALI in mice.

### Release of pro-inflammatory cytokines in BALF-exosomes by different cells after ALI

**Figure 3:**
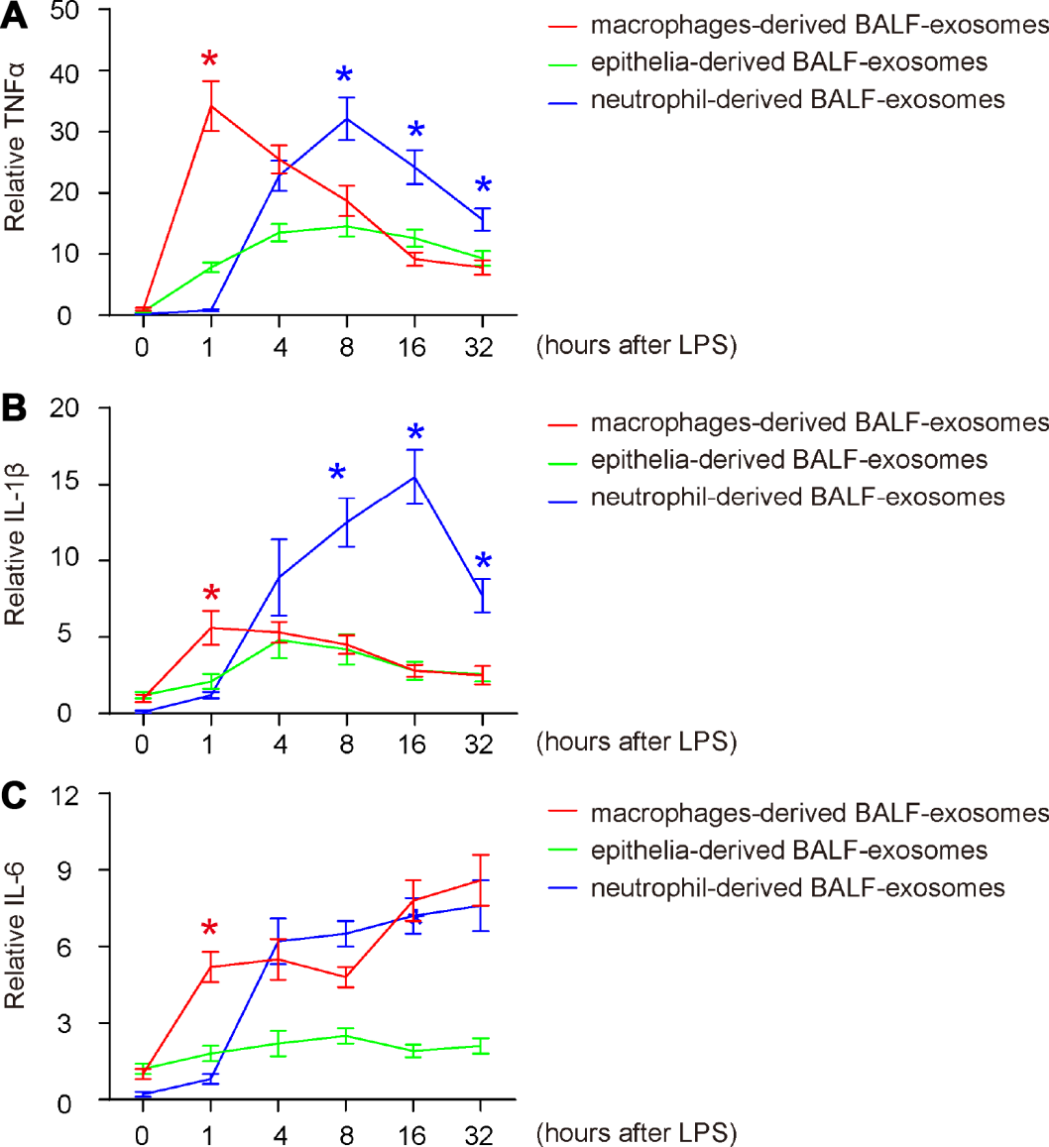
**Release of pro-inflammatory cytokines in BALF-exosomes by different cells after ALI.** (**A**–**C**) ELISA for 3 key pro-inflammatory cytokines (TNFα, IL-1β and IL-6) in respective BALF-exosomes at 1 hour, 4 hours, 8 hours, 16 hours and 32 hours after ALI. (**A**) TNFα. (**B**) IL-1β. (**C**) IL-6. *p<0.05. N=5.

First, we examined the release pattern of three key pro-inflammatory cytokines (TNFα, IL-1β and IL-6) in BALF-exosomes by different cells after ALI. For TNFα, we found that it started to increase in macrophage-derived exosomes as early as 1 hour after ALI, suggesting that the initial responsive cells to LPS should be macrophages, and their prompt release of TNFα suggests that they may be polarized from naïve macrophages to M1 macrophages (Figure 3A). Moreover, this early release of TNFα also appeared to be the peak value by macrophages, since TNFα derived from macrophages continuously decreased afterward (Figure 3A). The TNFα by epithelia appeared to increase from 1 hours after ALI, and likely peaked at 8 hours after ALI, with a relatively slow altering pattern (Figure 3A). On the other hand, The TNFα by neutrophils increased from 4 hours after ALI, but increased dramatically and appeared to be the major source of TNFα after 8 hours post ALI (Figure 3A). Together, these data suggest that LPS may first trigger for M1 polarization of macrophages, which release TNFα to recruit and differentiate neutrophils to further produce and secrete TNFα to mediate the early pro-inflammatory effects after ALI. Similar pattern was detected for IL-1β (Figure 3B), except for that its absolute increase, especially the first phase increase by macrophages, was modest (Figure 3B). The decrease of IL-1β after peak was also less pronounced (Figure 3B), and the peak of the modest increase of IL-1β by epithelia occurred at 4 hours after ALI (Figure 3B). For IL-6, its prompt release by macrophages did not decrease after 1 hour, while the contribution to IL-6 by macrophages and neutrophils was similar after 4 hours (Figure 3C). Thus, the pro-inflammatory cytokines in BALF-exosomes after ALI are derived primary from polarized M1 macrophages at early stage, and from neutrophils at later stages.

### Release of anti-inflammatory cytokines in BALF-exosomes by different cells after ALI

**Figure 4:**
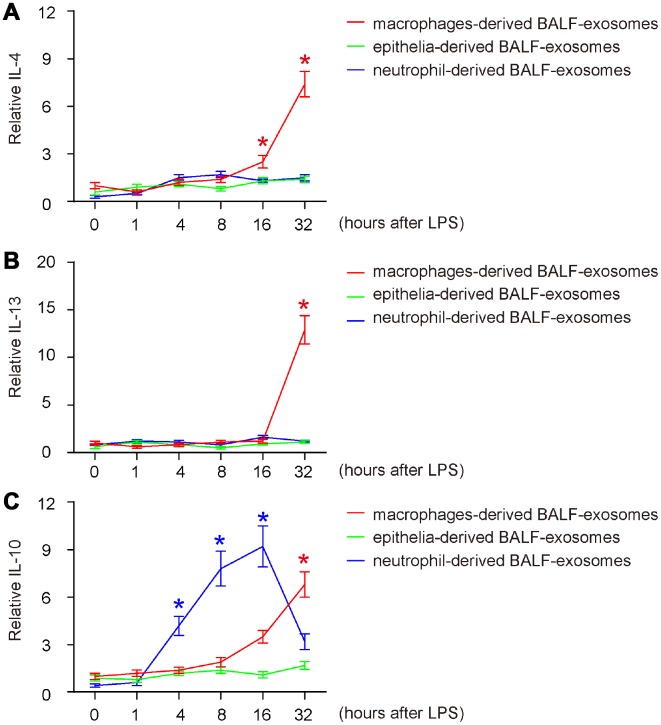
**Release of anti-inflammatory cytokines in BALF-exosomes by different cells after ALI.** (**A**–**C**) ELISA for 3 key anti-inflammatory cytokines (IL-4, IL-13 and IL-10) in respective BALF-exosomes at 1 hour, 4 hours, 8 hours, 16 hours and 32 hours after ALI. (**A**) IL-4. (**B**) IL-13. (**C**) IL-10. *p<0.05. N=5.

Next, we examined the release pattern of three key anti-inflammatory cytokines (IL-4, IL-13 and IL-10) in BALF-exosomes by different cells after ALI. For IL-4 and IL-13, we detected similar release patterns (Figure 4A, 4B). We found that both IL-4 and IL-13 started to increase at very late stage after ALI (IL-4 as late as 16 hours, and IL-13 as late as 32 hours) and were exclusively released by macrophages (Figure 4A, 4B). These data are consistent with established knowledge that both IL-4 and IL-13 are produced and secreted by T-cells and macrophages, but not by other cell types. The late release of IL-4 and IL-13 by BALF-macrophages suggests that they may be produced and secreted by polarized M2 macrophages, possibly in an autocrine manner to regulate macrophage phenotypic alternation to obtain appropriate functions for tissue repairing. While IL-10 was hardly released by epithelia, the earliest release of IL-10 was detected in BALF-exosomes derived from neutrophils at 4 hours after ALI without decrease till 16 hours (Figure 4C). On the other hand, the earliest release of IL-10 by macrophages appeared at 16 hours after ALI and further increased afterwards (Figure 4C). Since IL-10 is a well-known potent trigger of fibrotic macrophages- M2c, these data suggest that the differentiation of the fibrotic macrophage subtype M2c may be initiated by neutrophils-derived IL-10, and later by IL-10 derived from differentiated macrophages themselves.

### Release of fibrotic cytokines in BALF-exosomes by different cells after ALI

**Figure 5:**
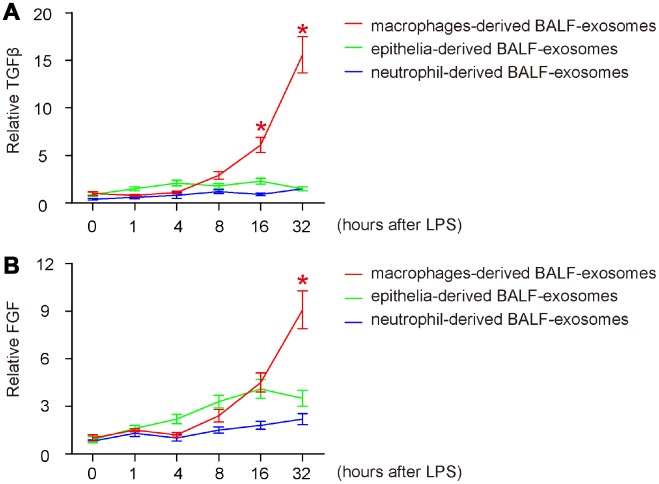
**Release of fibrotic cytokines in BALF-exosomes by different cells after ALI.** (**A**, **B**) ELISA for 2 key fibrotic cytokines (TGFβ and FGF) in respective BALF-exosomes at 1 hour, 4 hours, 8 hours, 16 hours and 32 hours after ALI. (**A**) TGFβ. (**B**) FGF. *p<0.05. N=5.

**Figure 6:**
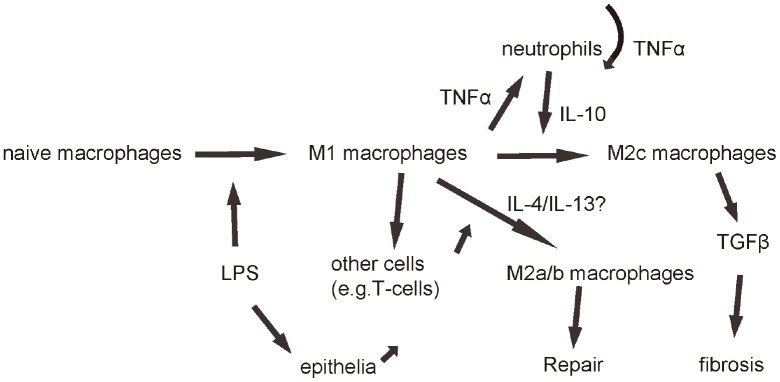
**Schematic of the model.** LPS first induces macrophage polarization to M1, which produces and releases TNFα to recruit and activate neutrophils, which further produced and secreted TNFα in an autocrine manner to mediate the early pro-inflammatory responses after ALI. Neutrophils also produce and secrete IL-10, which signals back to macrophages to polarize them to M2c to secrete TGFβ to mediate fibrosis.

Finally, we examined the release pattern of two key fibrotic cytokines (TGFβ and FGF) in BALF-exosomes by different cells after ALI, since they are related to outcome of post-ALI tissue repair and ARDS in human. For both, we detected similar release pattern like IL-10 (late release exclusively by macrophages), which is consistent with the knowledge that they are mainly M2c cytokines (Figure 5A, 5B), suggesting that the early release of IL-10 by neutrophils may be primary responsible for differentiating M2c, while the late release of IL-10 by polarized macrophages may be the main trigger for their production and secretion of TGFβ and FGF. However, compared to the nearly exclusive release of TGFβ by macrophages (Figure 5A), FGF was partially released by epithelia, as early as 4 hours after ALI (Figure 5B), suggesting that it may be from multisources. Hence, IL-10 is the initial trigger of fibrosis, and it may function through activating TGFβ- and FGF-secreting M2c macrophages. Our findings were then summarized in a schematic (Figure 6).

## DISCUSSION

One of the severe outcomes of ALI is ARDS, which is characterized by chronic inflammation in the airways or alveoli [8]. The most important cell types that contribute to the pathogenesis of ALI are macrophages, epithelial cells and neutrophils, compared to other cell types including T-cells, endothelial cells and mesenchymal cells [9]. It is obvious that the relationship among these cell types during ALI is interactive and dynamic, while one cell type “instructs” another cell type to alter its phenotype, which in turn talks back. The messages that are transferred among these cells in the microenvironment (BALF) are assembled into some small cargos, e.g. exosomes, to be released from one cell into BALF and then obtained by another to decipher the signals [10, 11]. Previous studies have not systemically analyzed the exosomes-cytokines from different types of cells in the microenvironment, although it is very important to understand the crosstalk among different cell types in ALI-associated immunology.

Here, we used a flow cytometry-based method to address this question. Co-expression of CD11c and F4/80 was used to determine macrophage-derived exosomes. CD11c is an integrin alpha X chain protein, functioning in the adherence of neutrophils and monocytes to stimulated endothelium cells, and in the phagocytosis of complement coated particles. CD11c is a marker for monocytes and macrophages, but also expressed in dendritic cells [12, 13]. F4/80 is EGF-like module-containing mucin-like hormone receptor-like 1 (EMR1), a member of the adhesion G protein-coupled receptor family. EMR1 expression in human is restricted to eosinophils and in mice is nearly restricted to macrophages [14]. Thus, co-expressing CD11c and F4/80 faithfully determined the exosomes from macrophage population. Also, that is the reason that we detected a few CD11c+F4/80- exosomes, which may be derived from dentritic cells. On the other hand, we used Epithelial cell adhesion molecule (EpCAM), a transmembrane glycoprotein mediating Ca2+-independent homotypic cell-cell adhesion in epithelia, as a unique marker for determining epithelia-derived exosomes [15]. Finally, co-expressing CD11b and Ly6G was used to determine neutrophils-derived exosomes, since CD11b is expressed by monocytes/macrophages, neutrophils, granulocytes, macrophages, and natural killer cells [16], while Ly6G is nearly exclusively expressed by neutrophils [17]. The CD11b+Ly6G- population should be mainly macrophages-derived exosomes.

The first interesting finding is TNFα, as it is the most important pro-inflammatory cytokine involved in the early pathogenesis of ALI. The major sources for TNFα are macrophages and neutrophils, while macrophages contribute to most early TNFα, neutrophils secreted most TNFα after 4 hours. Thus, it may be deducted that the early TNFα by macrophages should be secreted by the early macrophages responsive to the ALI-causes, LPS, and should be the trigger for all later responses. It is well-known that TNFα is a M1 macrophage cytokine [18], and thus the release of TNFα from macrophages at 1 hour after ALI should be from newly polarized macrophages, M1. The second wave of TNFα came from neutrophils, and it was clear that these neutrophils were activated later than those M1 macrophages, and were probably just activated by them, and even through TNFα [19].

The second important finding is IL-10/TGFβ. IL-10 is a known stimulator of M2c, the M2 macrophage subtype characterized with TGFβ production and fibrosis [20]. IL-10 was initially found to be released from neutrophils during ALI, which was a novel finding, since it was believed that either macrophages or T-cells may be the major source of IL-10 [21]. Here, our data showed very pronounced high IL-10 in the BALF-exosomes derived from neutrophils, consisting with some previous reports [22, 23], which suggests that neutrophils may be a major regulator for the differentiation of macrophages into M2c subtype that contributes to fibrosis through producing and secreting TGFβ. Although IL-10 may be also secreted by macrophages (apparently the case for IL-4 and IL-13 here), the high amount of IL-10 detected from neutrophils and the relative importance of neutrophils in ALI inflammation suggest that the effects of neutrophils are non-negligible here. It may be interesting to test whether blocking neutrophil-derived IL-10 could suppress M2c polarization of macrophages and thus reduce fibrosis post ALI.

To summarize, here we showed novel information on the precursor cells responsible for exosome-cytokines after ALI, and demonstrate an interactive and dynamic crosstalk among major cell types in the inflammatory response during ALI.

## MATERIALS AND METHODS

### Animals and protocols

All mouse experiment protocols were approved by the Animal Research and Care Committee at Tongji University. All experiments were performed in accordance with the guidelines from the Animal Research and Care Committee at Tongji University. Specific pathogen free (SPF) Balb/c mice (aged 10 weeks, weight 20g) were supplied by Shanghai Model Organisms Center (Shanghai, China). Mice were anaesthetised (intraperitoneal ketamine 90 mg/kg; xylazine 10 mg/kg), and 20 μg ‘ultrapure’ LPS (Sigma-aldrich, St Louis, MO, USA) in 50 μL was instilled intratracheally (i.t.). After 1 or 4 or 8 or 16 or 32 hours, animals were euthanised and tracheostomised to obtain BALF specimens.

### Permeability index and analysis of BALF-exosomes

For retrieval of BALF, airways were flushed with 0.8 ml PBS. The permeability index was determined in BALF collected at different time points after ALI. Permeability index is a quantitative marker for vascular leakage. BALF samples were recovered, followed by isolation of BALF-exosomes with total exosome isolation kit (Catalog number: 4478359; Invitrogen), which applies sequential centrifugation at 200g, 5 minutes and 4^o^C to remove cells and large particles, and at 20000g, 30 minutes and 4^o^C to isolate exosomes. Visualization of exosomes was done by electric microscopy and expression of CD9, CD63, CD81, ALIX and TSG101 was detected by RT-qPCR. Purified BALF-exosomes were then incubated with fluorescence-conjugated antibodies against CD11c/F4/80 (to identify alveolar macrophage-derived exosomes), EpCAM (to identify epithelial cell-derived exosomes) or CD11b/Ly6G (to identify neutrophil-derived exosomes), and then analysed by flow cytometry. The absolute counts for corresponding exosomes were assessed using counting beads.

### Quantitative RT-PCR

Total RNA was extracted by RNeasy kit (Qiagen, Valencia, CA, USA). Total RNA is transcribed by Omniscript reverse transcription kit (Qiagen) to generate complementary DNA (cDNA), which was used as templates in RT-qPCR. The primers used for RT-qPCR were also purchased from Qiagen. A 2-ΔΔCt method was used for quantification of the relative mRNA levels, and the values of gene expression were obtained by sequential normalization of the values to GAPDH and the experimental controls.

### ELISA

Total Protein was extracted using RIPA buffer (Sigma-Aldrich). Protein concentration was determined using a BCA kit. ELISA for mouse TNFα, IL-1β, IL-6, IL-4, IL-10, IL-13, TGFβ and FGF used ELISA kits (R&D Systems, Los Angeles, CA, USA), according to the manufacturer’s instruction.

### Statistical analysis

All values represent the mean ± standard deviation (SD). Statistical analysis was carried out using a two-way analysis of variance (ANOVA) test followed by the Fisher’s Exact Test to compare two groups using GraphPad Prism 7 (GraphPad, Chicago, IL, USA) software. A value of p<0.05 was considered statistically significant after Bonferroni correction.
